# The Effects of Trifluoroacetic Acid (TFA) in Humans: A Rapid Review

**DOI:** 10.3390/life15121825

**Published:** 2025-11-28

**Authors:** Jörg Wipplinger, Lukas Meusburger, Elisabeth Dottolo, Sonia Galazka, Lena Brunner, Alice Füreder, Viktoria Kundratitz, Karin Rainer, Elke Rauscher-Gabernig, Marlene Sarka, Johannes Pleiner-Duxneuner

**Affiliations:** AGES—Austrian Agency for Health and Food Safety, Spargelfeldstraße 191A, 1220 Vienna, Austria; lukas.meusburger@ages.at (L.M.); elisabeth.dottolo@ages.at (E.D.); sonia-ewa.galazka@ages.at (S.G.); alice.fuereder@ages.at (A.F.); elke.rauscher-gabernig@ages.at (E.R.-G.);

**Keywords:** trifluoroacetic acid, TFA, halothane, PFAS, human, toxicity

## Abstract

Human studies involving exposure to trifluoroacetic acid (TFA) and the associated clinical outcomes are typically not considered in standard chemical toxicity assessments. This review aimed to identify and synthesize all available human data on TFA exposure, regardless of study design or context. Given TFA’s long-standing use and its formation as a degradation product of various compounds, a wide range of exposure scenarios was considered, including post-anesthesia monitoring, environmental assessments, and chemical incidents. The database searches in MEDLINE (PubMed) and EMBASE (Scopus) were conducted on 28 and 29 April 2025. A total of 17 studies met the inclusion criteria: 4 case reports, 3 case series, 5 observational studies, and 5 pharmacokinetic studies. All studies documented clear human exposure to TFA and reported at least one TFA-related outcome. Some acute exposures exceeded the currently proposed threshold values. However, no study demonstrated clinically relevant effects attributable to TFA. As a strong acid, TFA can cause typical corrosive injuries upon direct contact, but no additional systemic or organ-specific toxic effects were observed.

## 1. Introduction

Trifluoroacetic Acid (TFA) is a short-chain perfluorocarboxylic acid and the final degradation product of many fluorinated compounds, such as per- and polyfluorinated alkyl substances (PFAS), pesticides, refrigerants, and fluorinated pharmaceuticals. TFA is rapidly absorbed through food intake [1], partially enters the enterohepatic circulation [2], is distributed throughout the body (including the placenta), and is excreted via urine and feces [1,3]. The toxicokinetic properties of this compound in humans have not been studied in detail. The acute toxicity of concentrations of TFA found in the environment is very low [1]. TFA is a component or degradation product of medical devices and pharmaceuticals, such as anesthetic gases. Models, cell studies, and animal studies are used to assess the toxicity of a substance, while clinical studies in humans usually do not play a role. Since TFA has been accumulating in the environment for several years [4] and, for many years, has come into direct contact with humans through medical interventions, this review will investigate whether there are studies examining the direct effects of any TFA exposure in humans. Due to these exposures, there is a possibility that clinical trials may uncover warning signs and risks that have been overlooked in previous assessments. Outcomes can include both clinical and laboratory parameters.

Our research question, based on the Population/Exposure/Comparator/Outcome (PECO) framework, is: What effect does Trifluoroacetic Acid (TFA) have in humans?

If this first research question confirms effects from TFA, our secondary research question would be: Is there a dose–response relationship of Trifluoroacetic Acid (TFA) in humans?

During the preparation of this review, the European Food Safety Authority (EFSA) launched its public consultation on a draft statement on consumer health-based guidance values on TFA [5]. For its new assessment, EFSA also considered human studies; however, compared to the present review, it conducted a less extensive search and consequently identified and included fewer studies.

## 2. Materials and Methods

This rapid review was conducted in accordance with the Cochrane rapid review methods guidance [6,7] and in line with the Preferred Reporting Items for Systematic Reviews and Meta-Analyses (PRISMA) [8]. This review was conducted in accordance with an a priori protocol [9] and has been registered in: https://doi.org/10.17605/OSF.IO/DY47W.

### 2.1. Inclusion and Exclusion Criteria

The following inclusion criteria were applied:

- Population: This review considered studies on humans, without restriction on age, sex, or health condition.
- Exposure: This review considered studies on explicit and certain exposure to TFA, also if it is formed as a metabolite of fluorinated pharmaceuticals (e.g., halothane) or as a degradation product of halogenated refrigerants and solvents, such as chlorofluorocarbons (CFCs) and hydrofluorocarbons (HFCs).
- Comparator (optional): This review considered studies in which humans without exposure to TFA were the comparators.
- Outcome: This review considered studies with any clinical or laboratory outcome investigated.

This rapid review considered primary studies, including case series, case reports, observational studies with comparison groups, non-randomized controlled trials, and randomized controlled trials. Reviews, opinions, and all other study types except primary studies were considered by this rapid review only as background literature, but not for the results.

### 2.2. Search Strategy

The search strategy aimed to find published primary studies on the effects of trifluoroacetic acid on humans.

We kept the search strategy wide to find any primary study examining the effects of TFA on humans. We made sure that predefined key papers were found by the developed search strategy. We made small adaptations to reduce the number of hits while still finding the predefined key papers.

The database search was conducted on 28 and 29 April 2025.

The full electronic search for MEDLINE (PubMed) resulted in:

(“human *” [Title/Abstract] OR “women” [Title/Abstract] OR “woman” [Title/Abstract] OR “men” [Title/Abstract] OR “man” [Title/Abstract] OR “child *” [Title/Abstract] OR “adolescen *” [Title/Abstract]) AND (“Trifluoroacetic” [Title/Abstract] OR “Trifluoracetic” [Title/Abstract] OR “trifluoracetate” [Title/Abstract] OR “Perfluoroacetic acid” [Title/Abstract] OR “F3CCOOH” [Title/Abstract] OR “CF3CO2H” [Title/Abstract] OR “F3CCO2H” [Title/Abstract] OR “cf3c o oh” [Title/Abstract] OR “CF3COOH” [Title/Abstract] OR “76-05-1” [EC/RN Number] OR “trifluoro acetic acid” [Title/Abstract] OR “trifluoro-acetic” [Title/Abstract] OR “Trifluoroacetic Acid” [MeSH Terms])

The full search strategies are provided in Appendix A.1 and Appendix A.2.

Studies published in English, German, and French were included. Other languages were not considered. No restriction was made on the year of publication.

The following databases were used: MEDLINE (PubMed) and EMBASE (Scopus).

Gray literature, such as the ECHA report, as well as internal reports from BfR and AGES, were considered for background context. They were provided to us by the experts involved in this review. We did not conduct any further gray literature searches.

### 2.3. Assessment of Study Quality

Of the 17 included studies, 4 were case reports, 3 case series, 5 observational studies, and 5 pharmacokinetic studies. The quality of the studies was assessed with the following tools:

Case reports: The JBI Critical Appraisal Checklist for Case Reports [10] comprises eight items with response options: ‘yes’, ‘no’, ‘unclear’, and ‘not applicable’. The checklist provides three possible outcomes, typically categorized as “Include”, “Exclude”, or “Seek further information”. In this review, we interpret these outcomes as indicators of three levels of methodological quality: “Include” corresponds to high quality, “Seek further information” to moderate quality, and “Exclude” to low quality.

Case Series: The JBI Critical Appraisal Checklist for Case Series [11] comprises ten items with response options: ‘yes’, ‘no’, ‘unclear’, and ‘not applicable’. The outcome measures are identical to those used in the checklist for case reports and were applied analogously in our evaluation.

Observational studies: The Newcastle–Ottawa Scale (NOS) is a tool for assessing the quality of non-randomized studies in meta-analyses [12]. It evaluates three domains: (1) *Selection* of study groups (representativeness of the exposed cohort, selection of the non-exposed cohort, ascertainment of exposure, and demonstration that outcome of interest was not present at baseline); (2) *Comparability* of cohorts based on design or analysis; (3) *Outcome* assessment (method of outcome determination, adequacy of follow-up duration, and completeness of follow-up). Each item is rated using a star system to reflect the risk of bias.

Pharmacokinetic studies: While instruments for evaluating the quality of pharmacokinetic studies are available, the substantial methodological heterogeneity and the age of several included studies rendered it more appropriate to rely on expert assessment; an experienced staff member of the Austrian medical market surveillance authority reviewed the studies for potential inconsistencies and methodological flaws. This approach ensured a qualified judgment regarding the methodological soundness and the plausibility of both the results and their presentation.

The quality assessment was performed by one author and was reviewed by a second author, except for the pharmacokinetic studies.

### 2.4. Data Extraction

As expected, a wide range of study types were included, from pharmacokinetic studies to observational studies. For data extraction, an Excel sheet was developed with the aim of capturing each exposure, dose, and clinical outcome. In addition, study type and study quality were documented. Data extraction was performed by one author and randomly checked by a second author.

### 2.5. Calculation of Exposure

In studies where TFA exposure results from the metabolism of fluorinated pharmaceuticals, we attempted to estimate total exposure based on the available data. The calculation of the TFA dose for study participants required various approaches. In several studies [13,14,15,16], TFA was quantified in urine following halothane exposure during anesthesia, with urine collection periods ranging from 3 to 15 days. Total TFA doses (mg/kg body weight) were determined using the aggregate amount of TFA excreted via urine and the mean body weight reported in each study. In the study by Monté et al. (1994) [17], TFA was measured after exposure to 1,1,1,2-tetrafluoroethane (HFA-134a) [17]. To estimate the total TFA dose of a most probable scenario, a lower bound approach was taken where TFA concentrations in urine below the limit of quantification (10 ng/mL) were approximated as zero. For the “worst-case” scenario, an upper-bound approach was chosen, where values below 10 ng/mL were set at that value. Nevertheless, only urine up to 6 h after exposure was analyzed, which might have led to a significant underestimation of the total TFA exposure. 

In other studies [18,19,20], exposure to halothane or desflurane during anesthesia was estimated using the duration at Minimum Alveolar Concentration (MAC), presented as either MAChours or MACminutes. The MAC is the concentration of an anesthetic gas at which 50% of patients do not respond to a stimulus (Eger et al., 1965) [21]. One MAChour is the exposure to an anesthetic gas at the MAC for one hour, with MACminutes being the analog for one minute. Nickalls and Mapleson (2003) report the MAC for halothane as 0.75% for adults and 0.9% for children, while the MAC for desflurane is estimated at 6.6% for adults [22]. The percentage of halothane absorbed, as noted in the study by Wark et al. (1990), in six children was 27% (see ANNEX) [15]. As a “worst-case” scenario, it was assumed that 50% of halothane was absorbed. The absorption of desflurane is assumed to be much lower, at around 8% (Buchinger et al., 2006) [23]. The inhalation rates for the study by Moore et al. 1986 [19] on twenty children were estimated from the study by Wark et al. (1990) [15]. The average minute ventilation rate of 206 mL kg^−1^ min^−1^ was used for the “most-probable” and the highest reported rate of 312 mL kg^−1^ min^−1^ for the “worst-case” scenario. For the adult patients in Bentley et al. 1981 [18] and Moore et al. 1986 [19], the inhalation rates were estimated using a respiratory rate of 12 breaths per minute and tidal volumes of 10 mL kg^−1^ for the “most-probable” and 15 mL kg^−1^ for the “worst-case” scenario. If a study did not report the body weight of the participants, a default value of 70 kg was used for adults. In studies with children, the body weights were always reported. According to the literature, the rate of metabolization of halothane to TFA is reported to be 12% with some estimates going as high as 20% [15,24,25]. These values were used to estimate the “most-probable” and “worst-case” scenario, respectively. In contrast, the metabolism of desflurane is considerably lower, with estimations being at 0.002% metabolization. 

In two studies, halothane concentrations were monitored in the air of operating rooms [26,27]. Wegner et al. (1990) reported the mean duration of exposure during the study [27]. This enabled the estimation of the total TFA dose received by each study group over the entire study period. For the “most-probable” scenario, the absorption of 27% and metabolism rate of 12% for TFA were assumed, while the “worst-case” scenario was estimated with 50% absorption and 20% metabolism. Additionally, the inhalation rate was estimated using the long-term exposure values for the age group 31 to <41 years given in the EPA Exposure Factors Handbook [28]. For the “most-probable” scenario, the mean rate of 16.0 m^3^/day was used, and the P95 of 21.2 m^3^/day for the “worst-case” scenario. For the study by Schaffernicht et al. [26], only the minimum and maximum halothane concentrations measured in the air were provided, and no duration of exposure was given. Therefore, the mean concentration was used to calculate the “most-probable” exposure scenario, while the maximum concentration was used for the “worst-case” scenario. The average exposure duration was estimated from the average exposure durations provided in [27]. Since all participants in both studies were adults, a default body weight of 70 kg was used for estimation purposes. More details are given in the Supplementary Material.

## 3. Results

### 3.1. Article Selection

Following the search, all references were exported from the databases to EndNote x9 (PubMed = 842 and Scopus = 1.866), where 395 duplicates were found by the program and removed by hand.

After, 2333 references were exported from EndNote to Rayyan after deduplication. Rayyan detected further duplicates within the set, and those 20 duplicates were removed.

Before starting the Title/Abstract screening on the remaining 2.313 references, we conducted a pilot exercise to ensure the consistent selection of articles for inclusion. For this pilot exercise, we created a random sample group of 5% of the 2.313 references) [29].

The 115 references were screened by 10 reviewers, who reached 97% agreement; therefore, the eligibility criteria were not modified. The three conflicts during the pilot screening round were resolved by a third party.

**Figure 1:**
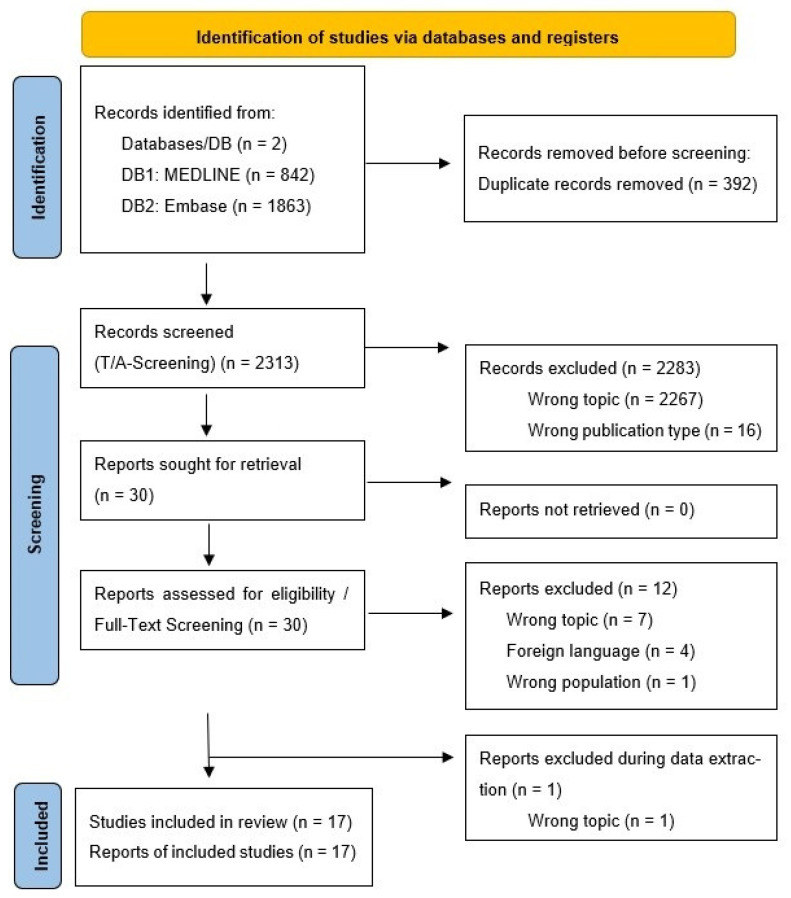
Search results, study selection, and inclusion process [8].

The 10 reviewers conducted the Title/Abstract screening for the remaining references. Conflicts were resolved by a third party. In total, 30 references were included and retrieved for full-text screening, which was conducted by two reviewers. Any conflicts at this stage were resolved through discussion (see Figure 1).

### 3.2. Description of the Studies Included

A total of 17 studies were included, comprising 4 case reports, 3 case series, 5 observational studies, and 5 pharmacokinetic studies (see Table 1). Some of the case reports are relatively recent, whereas most of the other studies date back more than 30 years. The most recent observational study was published in 1991.

### 3.3. Description of Outcomes Measured

The characteristics of the studies and their outcomes are presented in Table 1, Table 2 and Table 3, categorized by general description, type of exposure and outcome, and dose or total exposure. A total of 14 studies investigated a single exposure to TFA (acute), whereas only three studies involved prolonged or repeated exposure (chronic) (see Table 2).

#### 3.3.1. Types of Exposure

The included studies address various forms of TFA exposure: via inhalation, through anesthetic agents, such as halothane and desflurane, or through experimental inhalation (hydrochlorofluorocarbons, HFA-134a); chronic dermal contact in a laboratory setting; and skin exposure during acid-related accidents (see Table 2).

#### 3.3.2. Total TFA Exposure and Dose

**Figure 2:**
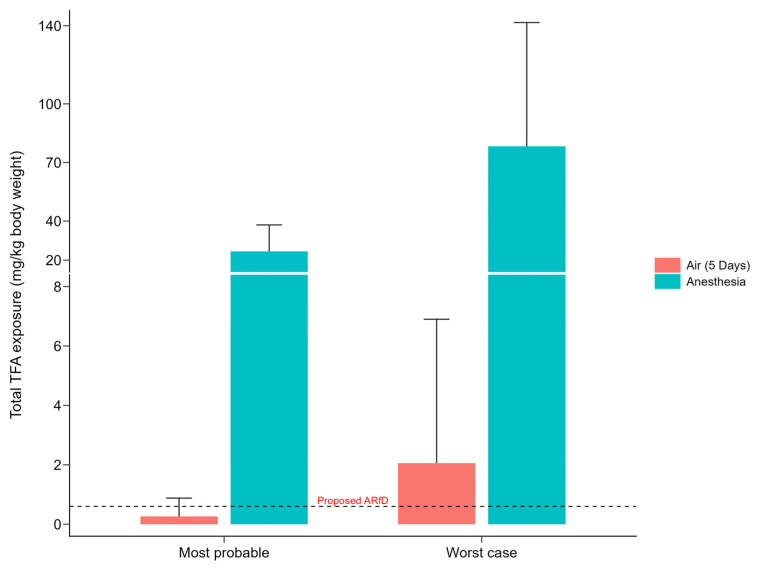
Total TFA exposure in halothane-related studies in (1) a most probable and (2) a worst-case scenario. The weighted mean + standard deviation of the TFA-exposure over the whole study duration, either from inhalation of air containing halothane in operating rooms or through halothane anesthesia, is shown. The weighted means were calculated based on the mean TFA exposures of each study, taking into account the number of participants for each study. The dotted line represents the Acute Reference Dose (ARfD) of 0.6 mg/kg body weight proposed by EFSA in its draft statement on consumer health-based guidance values on TFA [5].

As outlined in the Methods section, we attempted to calculate the total amount of TFA absorbed. Since the values for absorption rate and TFA metabolism are either unknown or not fully clarified, we calculated two scenarios: one based on likely estimates and one based on worst-case assumptions. The results are presented in Table 3; Figure 2 provides a summary of the halothane-related studies along with a comparison to the Acute Reference Dose (ARfD) of 0.6 mg/kg body weight proposed by the EFSA. The ARfD is defined as “an estimate of the amount of a substance in food and/or drinking water, normally expressed on a body weight basis that can be ingested in a period of 24 h or less, without appreciable health risk to the consumer, on the basis of all the known facts at the time of the evaluation” [37].

We have summarized the relevant clinical outcomes by type of exposure in Table 4. A more comprehensive table can be found in Appendix B.

## 4. Discussion

TFA has been accumulating in our environment for decades, and, due to its chemical properties, it is likely to persist for a long time to come [4]. It is therefore essential to assess the environmental impacts—and above all, the potential effects on human health—as quickly as possible. This review examined the extent to which the clinical effects of TFA in humans have been documented.

Overall, this review supports the current assessment of TFA as having low acute toxicity [2]; no new risks on humans were identified. Pharmacokinetic studies indicate accumulation in serum following repeated exposure to halothane and show delayed excretion of TFA, as it is partially subject to enterohepatic circulation [1].

Most clinical studies on TFA have been conducted in the context of halothane use. Halothane was used as an anesthetic for many years but is no longer in use due to reported cases of halothane-induced hepatitis, particularly in adults. These studies primarily describe the temporal course of halothane metabolism into TFA and other metabolites, as well as their detectability in blood and urine. Halothane as an anesthetic gas is no longer relevant; however, the doses of TFA produced through halothane metabolism provide an opportunity to assess the effects of very low doses, such as those currently absorbed from the environment. Although the route of exposure (inhalation versus ingestion via food or drinking water) introduces an additional source of uncertainty. One case report describes a child [33] who experienced liver failure upon first exposure to halothane. However, due to pre-existing conditions and additional risk factors, it is unlikely that TFA, as a halothane metabolite, contributed to the outcome. Halothane undergoes cytochrome P450-catalyzed oxidation to trifluoroacetic acid (TFA), bromide, and a reactive intermediate that can acetylate liver proteins. These protein neo-antigens stimulate an immune reaction that mediates severe hepatic necrosis (‘halothane hepatitis’) [38].

A clear distinction must be made between the systemic effects of low-dose exposure to TFA and the effects of TFA in its undiluted, strong acid form. The latter can cause local tissue damage due to its corrosive properties and is not representative of typical exposure scenarios in the general population. One included case series [34] not only illustrates the corrosive effects of TFA as a strong acid but also explores whether TFA may cause additional tissue damage, similar to what has been observed with hydrofluoric acid. For TFA, only the expected injuries typical of a strong acid were observed, with no other clinical effects, and subsequent blood tests were unremarkable. Only one case report from 2018 [30] described tissue damage that, according to the author, appeared more severe than what would have been expected from superficial acid burns alone.

Most of the included studies focus on single exposures, such as those occurring during anesthesia or accidents. Only three studies address repeated contact with TFA. The longest documented exposure is described in a case report [31], involving the development of dermatitis after more than 18 months of exposure to airborne TFA in a laboratory setting. Regarding repeated exposure among surgical personnel due to halothane use, we found only one study [27] in which TFA was mentioned. However, due to the lack of additional clinical endpoints, this study did not allow for any conclusions about whether long-term exposure poses a health risk to such personnel.

We did not find any studies that investigated long-term, chronic exposure to TFA. The years-long use of halothane as an anesthetic must have led to such exposure among surgical personnel, but we did not find any clinical studies that specifically analyzed the metabolite TFA in this context.

Figure 2 shows that patients receiving halothane anesthesia were exposed to total TFA doses well above the ARfD suggested by EFSA. In comparison, TFA exposure from inhaling halothane in operating room air was much lower but still exceeded the ARfD. However, it has to be noted that the ARfD is applied to oral exposure, while in this case, exposure was through inhalation. Additionally, exposure through room air was calculated for one work week (five days), while the ARfD is defined for up to one day. Therefore, comparison with the ARfD should not be interpreted as an assessment of potential health risks, but rather serves to illustrate the relative level of exposure.

One limitation of this review is that we did not include biomonitoring studies, but only studies with explicitly described TFA exposure. Such studies might potentially reveal the effects of elevated TFA levels, even though the source of exposure would remain unclear. Biomonitoring studies would require a dedicated systematic review to be addressed comprehensively. A publication bias cannot be ruled out; it is possible that studies indicating potential warning signals were not published. We do not consider the age of some studies as a limitation; rather, the comparison with halothane offers an opportunity to assess the effects of very low doses.

Both EFSA, in their draft statement on consumer health-based guidance values on TFA, and the German Federal Institute for Occupational Safety and Health (BAuA), in their CLH report submitted to ECHA CLH report—Proposal for Harmonised Classification and Labelling based on Regulation (EC) No 1272/2008 (CLP Regulation) [1], identified three human biomonitoring studies of TFA in humans. The first study quantified TFA in human serum with a detection rate of 97% with a median concentration of 8.46 ng/mL (n = 252 adults). Conversely, in the second study, TFA was analyzed in the urine of adolescents but not quantified. The detection rate was 30%, but the LOD was very high at 20 ng/mL. In the final study, the cord serum of 66 pairs of mothers and newborns was analyzed with a detection rate of 55% and a median concentration of 0.229 ng/mL [1]. However, the EFSA emphasized that these studies were not sufficient for risk assessment, and it was not possible to estimate the dietary intake or intake via the environment of TFA. In a newly published biomonitoring study investigating pooled urine samples from 6040 Australian individuals, TFA was detected in all samples, ranging from 3.4 to 300 µg/L, with a median concentration of 24 µg/L (Muir et al. 2025) [39]. Several European countries have set or proposed maximum limits for drinking water quality. In 2020, Germany’s Environment Agency derived a drinking water limit of 60 µg/L but recommended that TFA contents should remain below 10 µg/L. Similarly, Italy established a limit of 10 µg/L in 2025, while Denmark established a limit of 9 µg/L in 2021. In Belgium, Flanders enforces a provisional limit of 15.6 µg/L, whereas Wallonia has suggested a stricter limit of 2.2 µg/L. This value was adopted from a recommendation by the Dutch National Institute for Public Health and the Environment.

TFA is by far the most abundant PFAS in the environment [4]. According to data from the German Environment Agency, mean concentrations of TFA in precipitation have been reported at 0.335 μg/L [40]. TFA is widely detectable in groundwater across Austria. It was found in all samples taken from risk-based selected groundwater monitoring sites. The average TFA concentration is 0.71 µg/L, with a maximum value of 7.0 µg/L [41].

The toxicokinetics of TFA in humans have not yet been sufficiently investigated. Given its low acute toxicity at concentrations comparable to those currently found in drinking water, clinical studies would be feasible and could provide valuable insights into the effects of TFA within the human body.

## 5. Conclusions

The present work supports the view that TFA exhibits minimal acute toxicity. Its health effects in humans are primarily associated with its role as a metabolite in halothane-induced hepatitis and with chemical burns that may occur in accidents involving TFA in its form as a strong acid. However, this review cannot make any statements regarding the potential consequences of long-term, chronic, low-level exposure to TFA on human health. We recommend conducting a systematic review of biomonitoring studies and the clinical consequences of TFA exposure and incorporating these findings into future risk assessments.

## Figures and Tables

**Table 1:** Description of included studies.

Authors	Title	Publication Year	Study Type	Study Quality
D. H. Rochlin, C. M. Rajasingh, Y. L. Karanas et al. [30]	Full-Thickness Chemical Burn from Trifluoroacetic Acid: A Case Report and Review of the Literature	2018	Case Report	JBI Checklist for Case Reports: Include
J. Y. Byun, J. Y. Woo, Y. W. Choi et al. [31]	Occupational airborne contact dermatitis caused by trifluoroacetic acid in an organic chemistry laboratory	2014	Case Report	JBI Checklist for Case Reports: Include
C. Sun, B. Corbett [32]	Trifluoroacetic acid: Three times the fluoride, three times the toxicity?	2018	Case Report	JBI Checklist for Case Reports: Include
C. Nguyen, N. R. Rose, D. B. Njoku [33]	Trifluoroacetylated IgG4 antibodies in a child with idiosyncratic acute liver failure after first exposure to halothane	2008	Case Report	JBI Checklist for Case Reports: Include
H. Wark, J. Earl, D. Chau et al. [14]	Biliary excretion of the halothane metabolite trifluoroacetic acid in infants	1991	Case Series	(JBI) Critical Appraisal Checklist for Case Series: Seek further information (questions about methods are open)
J. Dahlin, M. Engfeldt, C. Svedman et al. [34]	Chemical burns caused by trifluoroacetic acid	2013	Case Series	(JBI) Critical Appraisal Checklist for Case Series: Include
P. Hoet, ML. M. Graf, M. Bourdi et al. [35]	Epidemic of liver disease caused by hydrochlorofluorocarbons used as ozone-sparing substitutes of chlorofluorocarbons	1997	Case Series	(JBI) Critical Appraisal Checklist for Case Series: Seek further information (questions about methods open)
J. B. Bentley, R. W. Vaughan, A. J. Gandolfi et al. [18]	Altered halothane metabolism: Obese vs. nonobese subjects	1981	Observational Study	Newcastle–Ottawa Scale (NOS): 6 of 9
R. Takiyama, M. Morio, K. Fujii et al. [13]	Clinical effects of halothane concentration on trifluoroacetic acid excretion in urine	1985	Observational Study	Newcastle–Ottawa Scale (NOS): 6 of 9
T. S. Sutton, D. D. Koblin, L. D. Gruenke et al. [20]	Fluoride metabolites after prolonged exposure of volunteers and patients to desflurane	1991	Observational Study	Not enough information for a NOS assessment
R. Wegner, B. Rincker, B. Poschadel et al. [27]	Halothane exposure of surgical staff in relation to the room air technology conditions	1990	Observational Study	Newcastle–Ottawa Scale (NOS): 6 of 9
R. A. Moore, K. W. McNicholas, J. D. Gallagher et al. [19]	Halothane metabolism in acyanotic and cyanotic patients undergoing open heart surgery	1986	Observational Study	Newcastle–Ottawa Scale (NOS): 5 of 9
E. Dallmeier, D. Henschler [36]	Determination and pharmacokinetics of trifluoroacetic acid after inhalation of low concentrations of halothane	1977	Pharmacokinetic study	Expert opinion: trustworthy
H. Schaffernicht, D. Kuchenbecker, J. Lehmann [26]	Determination of halothane in the urine of exposed persons as a method for biological exposure monitoring	1995	Pharmacokinetic study	Expert opinion: trustworthy
H. Wark, J. Earl, D. D. Chau et al. [15]	Halothane metabolism in children	1990	Pharmacokinetic study	Expert opinion: trustworthy
S. Y. Monté, I. Ismail, D. N. Mallett et al. [17]	The minimal metabolism of inhaled 1,1,1,2-tetrafluoroethane to trifluoroacetic acid in man as determined by high sensitivity 19F nuclear magnetic resonance spectroscopy of urine samples	1994	Pharmacokinetic study	Expert opinion: trustworthy
H. Wark, J. Earl, D. D. Chau et al. [16]	A urinary cysteine-halothane metabolite: validation and measurement in children	1990	Pharmacokinetic study	Expert opinion: trustworthy

**Table 2:** Description of studies: population, type of exposure, acute or chronic exposure and type of outcome.

Authors	N	Population	Form of Exposure	Duration	Outcome
D. H. Rochlin, C. M. Rajasingh, Y. L. Karanas et al. [30]	1	23-year-old healthy woman who worked as a chemist in a laboratory	dermal exposure	Acute	Healing of the wound, electrolyte testing
J. Y. Byun, J. Y. Woo, Y. W. Choi et al. [31]	1	24-year-old Korean man, healthy, job at an organic chemistry laboratory	airborne dermal contact	Chronic	Allergic contact dermatitis: patch tests
C. Sun, B. Corbett [32]	1	27-year-old male with no past medical history	dermal exposure	Acute	Distal sensation, strength, pulses, laboratory studies, telemetry monitoring, chemical burn evolution
C. Nguyen, N. R. Rose, D. B. Njoku [33]	1	4-year-old child tonsillectomy and adenoidectomy	halothane anesthesia	Acute	Acute liver failure and clinical course
H. Wark, J. Earl, D. Chau et al. [14]	2	infants of 2 and 5 months	halothane anesthesia	Acute	TFA in the bile
J. Dahlin, M. Engfeldt, C. Svedman et al. [34]	5	One 22-year-old male, 4 women between 29 and 48 years of age; all worked at the same medium-sized company, which uses trifluoroacetic acid in its production process	dermal exposure	Acute	Healing of the skin, blood levels, and liver levels
P. Hoet, ML. M. Graf, M. Bourdi et al. [35]	9	industrial workers	inhalation of hydrochlorofluorocarbons	Chronic	Acute hepatitis/liver disorders: blood biochemistry, liver biopsy sample
J. B. Bentley, R. W. Vaughan, A. J. Gandolfi et al. [18]	25	17 morbidly obese, 8 nonobese patients	halothane anesthesia	Acute	TFA Level in blood
R. Takiyama, M. Morio, K. Fujii et al. [13]	12	Japanese patients without history of blood transfusion, liver disease, general anesthesia, or drug injection; 6 low-halothane concentration, 6 high-halothane concentration	halothane anesthesia	Acute	TFA in urine
T. S. Sutton, D. D. Koblin, L. D. Gruenke et al. [20]	39	13 healthy volunteers, 26 healthy patients undergoing elective surgery	desflurane anesthesia	Acute	TFA in blood and urine
R. Wegner, B. Rincker, B. Poschadel et al. [27]	31	All operation staff (anesthetists, surgeons, nurses); 15 men, 16 women	inhalation of halothane	Both	TFA in urine
R. A. Moore, K. W. McNicholas, J. D. Gallagher et al. [19]	20	Patients with congenital heart disease scheduled for open-heart surgery	halothane anesthesia	Acute	TFA in blood
E. Dallmeier, D. Henschler [36]	6	Volunteers	inhalation of halothane	Acute	TFA in blood
H. Schaffernicht, D. Kuchenbecker, J. Lehmann [26]	13	5 anesthetists, 8 volunteers	inhalation of halothane	Acute	TFA in urine
H. Wark, J. Earl, D. D. Chau et al. [15]	6	Children with mean age of 74 months; 5 healthy, 1 with cystic fibrosis	halothane anesthesia	Acute	TFA in urine
S. Y. Monté, I. Ismail, D. N. Mallett et al. [17]	4	healthy male volunteers	inhalation of HFA-134a	Acute	TFA in urine
H. Wark, J. Earl, D. D. Chau et al. [16]	6	children; 5 healthy; 1 with cystic fibrosis, chronic lung disease, and proven liver disease	halothane anesthesia	Acute	TFA in urine

**Table 3:** Calculation of the most probable and the worst-case exposure to TFA in the included studies.

Authors	Most Probable	Worst Case
H. Wark, J. Earl, D. Chau et al. [14]	Patient 1: 29.3 mg/kg bw; Patient 2: 14.2 mg/kg bw	Not Applicable (NA)
J. B. Bentley, R. W. Vaughan, A. J. Gandolfi et al. [18]	Obese: 9.1 mg/kg bw; Nonobese: 9.4 mg/kg bw (body weight)	Obese: 42.3 mg/kg bw; Nonobese: 43.6 mg/kg bw
R. Takiyama, M. Morio, K. Fujii et al. [13]	Group 1 (low dose, long duration): 43 ± 6 mg/kg bw; Group 2 (high dose, short duration): 40 ± 10 mg/kg bw	NA
T. S. Sutton, D. D. Koblin, L. D. Gruenke et al. [20]	Volunteers: 2.6 mg/kg bw; Patients: 1.1 mg/kg bw	Volunteers: 12.2 mg/kg bw; Patients: 5.1 mg/kg bw
R. Wegner, B. Rincker, B. Poschadel et al. [27]	Anesthetists: 0.016 mg/kg bw; Surgeons: 0.008 mg/kg bw; Surgery staff: 0.007 mg/kg bw; Floaters: 0.004 mg/kg bw	Anesthetists: 0.064 mg/kg bw; Surgeons: 0.031 mg/kg bw; Surgery staff: 0.030 mg/kg bw; Floaters: 0.015 mg/kg bw
R. A. Moore, K. W. McNicholas, J. D. Gallagher et al. [19]	Acyanotic: 36 mg/kg bw; Cyanotic: 37 mg/kg bw	Acyanotic: 168 mg/kg bw; Cyanotic: 171 mg/kg bw
H. Schaffernicht, D. Kuchenbecker, J. Lehmann [26]	Anesthetists: 0.84 mg/kg bw; Volunteers 0.90 mg/kg bw	Anesthetists: 6.3 mg/kg bw; Volunteers 7.2 mg/kg bw
H. Wark, J. Earl, D. D. Chau et al. [15]	15.9 ± 3.6 mg/kg bw	NA
S. Y. Monté, I. Ismail, D. N. Mallett et al. [17]	Volunteer 1: 0 µg/kg bw; Volunteer 2: 0.023 µg/kg bw; Volunteer 3: 0.027 µg/kg bw; Volunteer 4: 0.068 µg/kg bw	Volunteer 1: 0.075 µg/kg bw; Volunteer 2: 0.041 µg/kg bw; Volunteer 3: 0.048 µg/kg bw; Volunteer 4: 0.102 µg/kg bw
H. Wark, J. Earl, D. D. Chau et al. [16]	15.9 ± 3.6 mg/kg bw (comparison halothane metabolism in children)	NA

**Table 4:** Summary of findings for clinical outcomes.

Exposure	Authors	Outcome
Airborne dermal contact	J. Y. Byun, J. Y. Woo, Y. W. Choi et al. [31]	Allergic contact dermatitis caused by TFA; in 5 healthy volunteers, patch test with TFA 1%, 0.1%, and 0.01% did not lead to any reactions
Dermal exposure	D. H. Rochlin, C. M. Rajasingh, Y. L. Karanas et al. [30]	First reported incidence of a chemical burn due to TFA greater than 4% total body surface area (approx. 15%); consistent with the classic description of chemical burn (depth is underestimated) = burns initially appeared superficial but evolved to necrosis in one week. Discharged hospital day 35. Follow-up: Healed with no functional deficits
Dermal exposure	C. Sun, B. Corbett [32]	4% body surface area (BSA) circumferential partial thickness burn of the right forearm with a 0.5 cm by 2 cm area of hyperpigmentation
Dermal exposure	J. Dahlin, M. Engfeldt, C. Svedman et al. [34]	Burns healed as expected for chemical burns caused by acids. Collected blood samples were normal (liver status, creatinine, electrolytes)
Halothane anesthesia	C. Nguyen, N. R. Rose, D. B. Njoku [33]	Case of a 4-year-old with hepatic failure 15 days post-op (POD) → ICU, allowed to go home POD 21, healthy after 1 month
Inhalation of hydrochlorofluorocarbons	P. Hoet, ML. M. Graf, M. Bourdi et al. [35]	Liver biopsy sample showed hepatocellular necrosis; trifluoroacetyl-adducted proteins were detected in surviving hepatocytes. Autoantibodies against P450 2E1 or P58 were detected in the serum of five affected workers.

## Data Availability

No new data were created or analyzed in this study. Data sharing is not applicable to this article.

## Supplementary Materials

The following supporting information can be downloaded at: https://www.mdpi.com/article/10.3390/life15121825/s1, ANNEX.

## Appendix A

### Appendix A.1. Search Strategy PubMed—28 April 2025

**PECO Element**		**Search Terms + MeSH Terms**
Population	Humans	“human *” [Title/Abstract] OR “women” [Title/Abstract] OR “woman” [Title/Abstract] OR “men” [Title/Abstract] OR “man” [Title/Abstract] OR “child *” [Title/Abstract] OR “adolescen *” [Title/Abstract]
Exposure	Exposure to at least one of the TFA or the associated salts. There are no limitations on the timing, route, level, or determination of estimated exposure.	“TFA” [Title/Abstract] OR “Trifluoroacetic” [Title/Abstract] OR “Trifluoracetic” [Title/Abstract] OR “trifluoracetate” [Title/Abstract] OR “Perfluoroacetic acid” [Title/Abstract] OR “F3CCOOH” [Title/Abstract] OR “CF3CO2H” [Title/Abstract] OR “F3CCO2H” [Title/Abstract] OR “cf3c o oh” [Title/Abstract] OR “CF3COOH” [Title/Abstract] OR “76-05-1” [EC/RN Number] OR (“trifluoro acetic acid” [Title/Abstract]) OR (“trifluoro-acetic” [Title/Abstract]) OR “Trifluoroacetic Acid” [MeSH Terms]
Comparator	Humans	
Outcomes	Any health outcome or type of biological response.	

**Search Number**	**Query**
3	#1 AND #2
2	“Trifluoroacetic” [Title/Abstract] OR “Trifluoracetic” [Title/Abstract] OR “trifluoracetate” [Title/Abstract] OR “Perfluoroacetic acid” [Title/Abstract] OR “F3CCOOH” [Title/Abstract] OR “CF3CO2H” [Title/Abstract] OR “F3CCO2H” [Title/Abstract] OR “cf3c o oh” [Title/Abstract] OR “CF3COOH” [Title/Abstract] OR “76-05-1” [EC/RN Number] OR (“trifluoro acetic acid” [Title/Abstract]) OR (“trifluoro-acetic” [Title/Abstract]) OR “Trifluoroacetic Acid” [MeSH Terms]
1	“human *” [Title/Abstract] OR “women” [Title/Abstract] OR “woman” [Title/Abstract] OR “men” [Title/Abstract] OR “man” [Title/Abstract] OR “child*” [Title/Abstract] OR “adolescen*” [Title/Abstract]

### Appendix A.2. Search Strategy Scopus—29 April 2025

**Table:**

Search Number	Query
4	#1 AND #2 AND (LIMIT-TO (SUBJAREA, “BIOC”) OR LIMIT-TO (SUBJAREA, “PHAR”) OR LIMIT-TO (SUBJAREA, “MEDI”) OR LIMIT-TO (SUBJAREA, “AGRI”) OR LIMIT-TO (SUBJAREA, “IMMU”) OR LIMIT-TO (SUBJAREA, “NEUR”) OR LIMIT-TO (SUBJAREA, “HEAL”) OR LIMIT-TO (SUBJAREA, “MULT”) OR LIMIT-TO (SUBJAREA, “NURS”) OR LIMIT-TO (SUBJAREA, “SOCI”) OR LIMIT-TO (SUBJAREA, “DENT”)) AND (EXCLUDE (EXACTKEYWORD, “Nonhuman”) OR EXCLUDE (EXACTKEYWORD, “Animals”) OR EXCLUDE (EXACTKEYWORD, “Animal”) OR EXCLUDE (EXACTKEYWORD, “Animal Experiment”) OR EXCLUDE (EXACTKEYWORD, “In Vitro Study”) OR EXCLUDE (EXACTKEYWORD, “Rat”) OR EXCLUDE (EXACTKEYWORD, “Mouse”) OR EXCLUDE (EXACTKEYWORD, “Animal Tissue”) OR EXCLUDE (EXACTKEYWORD, “Mice”) OR EXCLUDE (EXACTKEYWORD, “Animal Cell”) OR EXCLUDE (EXACTKEYWORD, “Rats”) OR EXCLUDE (EXACTKEYWORD, “Animal Model”)) *
3	#1 AND #2
2	((TITLE-ABS-KEY (trifluoroacetic *) OR TITLE-ABS-KEY (trifluoracet *) OR TITLE-ABS-KEY (“Perfluoroacetic acid”) OR TITLE-ABS-KEY (“F3CCOOH”) OR TITLE-ABS-KEY (“CF3CO2H”) OR TITLE-ABS-KEY (“F3CCO2H”) OR TITLE-ABS-KEY (“cf3c o oh”) OR TITLE-ABS-KEY (“CF3COOH”) OR TITLE-ABS-KEY (“trifluoro acetic acid”) OR TITLE-ABS-KEY (“trifluoro-acetic”) OR CASREGNUMBER (76-05-1))
1	((TITLE-ABS-KEY (human) OR TITLE-ABS-KEY (wom?n) OR TITLE-ABS-KEY (m?n) OR TITLE-ABS-KEY (child *) OR TITLE-ABS-KEY (adolescen *)))

* The limitations were achieved by excluding certain keywords and subject areas in the filter.

## Appendix B. Compact Summary Table

**Authors**	**Study** **Design**	**Population**	**Form of Exposure**	**Most Probable**	**Worst Case**	**Outcome Measured**	**Study Quality**
D. H. Rochlin, C. M. Rajasingh, Y. L. Karanas et al. [28]	Case Report	23-year-old healthy woman who worked as a chemist in a laboratory	dermal exposure	-	-	Healing of the wound, electrolyte testing	JBI Checklist for Case Reports: Include
J. Y. Byun, J. Y. Woo, Y. W. Choi et al. [29]	Case Report	24-year-old Korean man, healthy, job at an organic chemistry laboratory	airborne dermal contact	-	-	allergic contact dermatitis: patch tests	JBI Checklist for Case Reports: Include
C. Sun, B. Corbett [30]	Case Report	27-year-old male with no past medical history	dermal exposure	-	-	distal sensation, strength, pulses, laboratory studies, telemetry monitoring, chemical burn evolution	JBI Checklist for Case Reports: Include
C. Nguyen, N. R. Rose, D. B. Njoku [31]	Case Report	4-year-old child tonsillectomy and adenoidectomy	halothane anesthesia	-	-	Acute liver failure and clinical course	JBI Checklist for Case Reports: Include
H. Wark, J. Earl, D. Chau et al. [14]	Case Series	infants of two and five months	halothane anesthesia	Patient 1: 29.3 mg/kg bw; Patient 2: 14.2 mg/kg bw	NA	TFA in the bile	(JBI) Critical Appraisal Checklist for Case Series- seek further information (questions about methods are open)
J. Dahlin, M. Engfeldt, C. Svedman et al. [32]	Case Series	One 22-year-old male, 4 women between 29 and 48 years of age; all worked at the same medium-sized company, which uses trifluoroacetic acid in its production process	dermal exposure	-	-	Healing of the Skin, blood levels, liver levels	(JBI) Critical Appraisal Checklist for Case Series: Include
P. Hoet, ML. M. Graf, M. Bourdi et al. [33]	Case Series	industrial workers	inhalation of Hydrochlorofluorocarbons	-	-	acute hepatitis/liver disorders: blood biochemistry, liver biopsy sample,	(JBI) Critical Appraisal Checklist for Case Series- seek further information (questions about methods open)
J. B. Bentley, R. W. Vaughan, A. J. Gandolfi et al. [18]	Observational Study	17 morbidly obese, 8 nonobese patients	halothane anesthesia	Obese: 9.1 mg/kg bw; Nonobese: 9.4 mg/kg bw (body weight)	Obese: 42.3 mg/kg bw; Nonobese: 43.6 mg/kg bw	TFA Level in blood	Newcastle–Ottawa Scale (NOS): 6 of 9
R. Takiyama, M. Morio, K. Fujii et al. [13]	Observational Study	Japanese patients without history of blood transfusion, liver disease, general anesthesia, or drug injection; 6 low halothane concentration, 6 high halothane concentration	halothane anesthesia	Group 1 (low dose, long duration): 43 ± 6 mg/kg bw; Group 2 (high dose, short duration): 40 ± 10 mg/kg bw	NA	TFA in urine	Newcastle–Ottawa Scale (NOS): 6 of 9
T. S. Sutton, D. D. Koblin, L. D. Gruenke et al. [20]	Observational Study	13 healthy volunteers, 26 healthy patients undergoing elective surgery	desflurane anesthesia	Volunteers: 2.6 mg/kg bw; Patients: 1.1 mg/kg bw	Volunteers: 12.2 mg/kg bw; Patients: 5.1 mg/kg bw	TFA in blood and urine	Not enough information for a NOS assessment
R. Wegner, B. Rincker, B. Poschadel et al. [26]	Observational Study	All operation staff (anesthetists, surgeons, nurses); 15 men, 16 women	inhalation of halothane	Anesthetists: 0.016 mg/kg bw; Surgeons: 0.008 mg/kg bw; Surgery staff: 0.007 mg/kg bw; Floaters: 0.004 mg/kg bw	Anesthetists: 0.064 mg/kg bw; Surgeons: 0.031 mg/kg bw; Surgery staff: 0.030 mg/kg bw; Floaters: 0.015 mg/kg bw	TFA in urine	Newcastle–Ottawa Scale (NOS): 6 of 9
R. A. Moore, K. W. McNicholas, J. D. Gallagher et al. [19]	Observational Study	Patients with congenital heart disease scheduled for open-heart surgery	halothane anesthesia	Acyanotic: 36 mg/kg bw; Cyanotic: 37 mg/kg bw	Acyanotic: 168 mg/kg bw; Cyanotic: 171 mg/kg bw	TFA in blood	Newcastle–Ottawa Scale (NOS): 5 of 9
E. Dallmeier, D. Henschler [34]	Pharmacokinetic study	Volunteers	inhalation of halothane	-	-	TFA in blood	Expert opinion: trustworthy
H. Schaffernicht, D. Kuchenbecker, J. Lehmann [25]	Pharmacokinetic study	5 anesthetists, 8 volunteers	inhalation of halothane	Anesthetists: 0.84 mg/kg bw; Volunteers 0.90 mg/kg bw	Anesthetists: 6.3 mg/kg bw; Volunteers 7.2 mg/kg bw	TFA in urine	Expert opinion: trustworthy
H. Wark, J. Earl, D. D. Chau et al. [15]	Pharmacokinetic study	Children with mean age of 74 months; 5 healthy, one with cystic fibrosis	halothane anesthesia	15.9 ± 3.6 mg/kg bw	NA	TFA in urine	Expert opinion: trustworthy
S. Y. Monté, I. Ismail, D. N. Mallett et al. [17]	Pharmacokinetic study	healthy male volunteers	inhalation of HFA-134a	Volunteer 1: 0 µg/kg bw; Volunteer 2: 0.023 µg/kg bw; Volunteer 3: 0.027 µg/kg bw; Volunteer 4: 0.068 µg/kg bw	Volunteer 1: 0.075 µg/kg bw; Volunteer 2: 0.041 µg/kg bw; Volunteer 3: 0.048 µg/kg bw; Volunteer 4: 0.102 µg/kg bw	TFA in urine	Expert opinion: trustworthy
H. Wark, J. Earl, D. D. Chau et al. [16]	Pharmacokinetic study	children; 5 healthy; one with cystic fibrosis, chronic lung disease and proven liver disease	halothane anesthesia	15.9 ± 3.6 mg/kg bw (comparison halothane metabolism in children)	NA	TFA in urine	Expert opinion: trustworthy
