# Supplementary material for: The Effects of Trifluoroacetic Acid (TFA) in Humans: A Rapid Review
Source: Life (Basel). 2025 Nov 28;15(12):1825. doi: 10.3390/life15121825 (PMC12733928; doi:10.3390/life15121825)
Supplement: Supplementary file 1 [file life-15-01825-s001.zip › life-3976339-supplementary.pdf]

ANNEX

C 1. The effects of Trifluoroacetic Acid (TFA) in humans: a rapid review

Assumptions used for TFA Exposure Estimation

Absorption of Halothane:

It was difficult to find literature data on the fraction of halothane absorbed. The most expansive data could be found in the study by (Wark et al., 1990), where five children were anesthetized with halothane for one hour. Using the minute ventilation, time of administration (*t*) and halothane absorption values provided in table II of the study as well as the fraction of halothane in the inspired gas mixture of 1% v/v (F_I), it was possible to make a rough estimation of the total amount of halothane administered (equation 1). Thereby, under the assumption of ideal gas behavior the molar volume of 25.45 L/mol at 25°C and a pressure of 1 atm (V_m) were applied, and a molar mass (*M*) for halothane of 197.39 g/mol. The time of halothane administration was described as 60 minutes for all patients except for patient number 1, where halothane was only administered for 55 minutes.

$$Total\ Halothane\ \left[\frac{mg}{kg}\right] = \frac{Minute\ ventilation\ \left[\frac{mL}{kg * min}\right] \cdot F_I \cdot t[min] \cdot 1000\ \left[\frac{mg}{g}\right]\ \cdot M\left[\frac{g}{mol}\right]}{V_m\ \left[\frac{L}{mol}\right]} \qquad (1)$$

Using the amount of halothane absorbed in mg/kg body weight for each patient, the fraction could be estimated using equation 2. The absorption fractions for each patient are given in Table S1. The mean of all estimations was used for the most probable scenarios.

$$Absorbed\ Fraction = \frac{Halothane\ Absorption\ \left[\frac{mg}{kg}\right] \cdot 100\%}{Total\ Halothane\ in\ ventilation\ \left[\frac{mg}{kg}\right]} \qquad (2)$$

Table S1: Minute ventilation and halothane absorption data provided in the study by (Wark et al., 1990). The total halothane in ventilation was calculated using equation 1 and the fraction of halothane absorbed was estimated using equation 2.

Patient No	Minute ventilation (mL kg^{-1} min^{-1})	**Total halothane in ventilation (mg/kg)**	Halothane absorption (mg/kg)	**Fraction of halothane absorbed (%)**
1	312	**1384.5**	270	**20%**
2	260	**1258.6**	310	**25%**
3	257	**958.0**	236	**25%**

4	107	**518.0**	176	**34%**
5	190	**919.8**	271	**29%**
5	142	**687.4**	226	**33%**
6	172	**832.6**	226	**27%**
Mean	206	**937.0**	245	**27%**
SD	68	**280**	40	**5%**

As a worst-case scenario an assumption of 50% halothane absorption was made, as the estimation of 27% absorption entailed several assumptions and uncertainties. Firstly, the sample size of only six patients was very small and only included children. Therefore, it is unclear whether these estimations can be reliably extrapolated to other age groups such as adults. Secondly, the estimation of the total halothane dose in the inspired gas mixture included the assumption of a pressure of 1 atm and a temperature of 25°C as well as assuming ideal gas behaviour, which might have led to an over- or underestimation of the total halothane inspired. Finally, urine was generally collected for seven or eight days postoperatively. However, in two patients it was only collected for three and four days, respectively, which might have led to an underestimation.

Metabolism of Halothane:

Regarding the metabolism of halothane, for the most probable scenario 12% metabolization was assumed due to the results of (Wark et al., 1990) and (Rehder et al., 1967), who found that on average 11.4% and 12% of the absorbed halothane was excreted in urine as TFA, respectively. As a worst case scenario, the values at the higher end mentioned on the website of the Institute for veterinary pharmacology and toxicology of the university of Zürich were used, which reported metabolization of halothane to TFA of 12-20% (Demuth & Müntener).

Absorption of Desflurane:

https://doi.org/10.1007/s00101-006-1059-7) on 26 patients, the absorption of desflurane was reported to be at around 8%.

Metabolism of Desflurane:

Desflurane has a very low metabolization rate which is estimated to be (Striebel Die Anästhesie Die Anästhesie von Hans W Striebel | ISBN 978-3-7945-2636-9 | Fachbuch online kaufen - Lehmanns.de. However, the specific proportion metabolized to TFA remains unclear, which may have led to a considerable overestimation of the actual amount of TFA produced.

Inhalation Rate – Anaesthesia Adults:

Traditionally, before 2000 high tidal volumes of around 10-15 mL kg^{-1}were used for mechanical ventilation during anaesthesia whilst nowadays normally lower tidal volumes are used (Marini et al 1996 https://doi.org/10.1016/S0272-5231(05)70331-2). As all studies were performed before 2000, these volumes were used for estimation of the inhalation rate. Assuming a respiratory rate of 12 breaths per minute, the

minute ventilation ranges from 120 to 180 mL kg^{-1} min^{-1}. For the "most-probable" scenario, 120 mL kg^{-1} $min^{-1}$was used as the estimate, while 180 mL kg^{-1} min^{-1} was applied for the "worst-case" scenario.

Inhalation Rate – Anaesthesia Children

The minute ventilation rates provided in the study by Wark et al. 1990 on six children were used for estimation of the inhalation rates of children with exposure to halothane during anaesthesia. The average minute ventilation of 206 mL kg^{-1} min^{-1} was used for the “most-probable” and the highest reported rate of 312 mL kg^{-1} min^{-1} for the “worst-case” scenario.

Inhalation Rate – Room Air

To estimate the TFA exposure through inhalation of halothane in room air, the long-term exposure values for the age group 31 to <41 years given in the EPA Exposure Factors Handbook (REF) were used. For the “most-probable” scenario the mean rate of 16.0 m^3/day was used and the P95 of 21.2 m^3/day for the “worst-case” scenario.

Table S2: List of assumptions used to estimate the TFA exposure for the “most-probable” and “worst-case” scenarios.

	Assumption	Most-probable	Source	Worst-case	Source
Halothane	Absorption Fraction	27%	Wark 1990	50%	
	Metabolism Fraction	12%	Rehder 1967, Wark 1990	20%	Uni Zürich Wirkstoff: Halothan - Pharmakokinetik
Desflurane	Absorption Fraction	8%	Buchinger	NA	
	Metabolism Fraction	0.002%	Striebel Die Anästhesie Band I Auflage 2 (2010)	NA	
Inhalation rates	Inhalation Rate - Anaesthesia Adults	120 mL kg^{-1} min^{-1}	Marini et al. 1996	180 mL kg^{-1} min^{-1}	Marini et al. 1996
	Inhalation Rate - Anaesthesia Children	210 mL kg^{-1} min^{-1}	Wark 1990	310 mL kg^{-1} min^{-1}	Wark 1990
	Inhalation Rate - Long-Term	16.0 m^3 day^{-1}	EPA Expo Factors Handbook	21.2 m^3 day^{-1}	EPA Expo Factors Handbook
Body weight	If no body weight was provided in the study, 70 kg were used for adults.				

Uncertainty Analysis:

Table S3: Uncertainty analysis for representative studies for one patient group per study. The most probable scenario was taken as the base assumption. For the new assumption only one parameter is changed. Δ indicates the difference between the new assumption to the base assumption.

		Base Assumption		New Assumption		
Study – group	Parameter	Most-probable	Exposure (mg/kg)	Worst-case	Exposure (mg/kg)	**Δ (mg/kg)**
Wegner et al. 1990 – Anesthetists	Absorption fraction	27%	0.016	50%	0.029	**0.013**
	Metabolism fraction	12%	0.016	20%	0.026	**0.010**
	Inhalation rate	16 m^3 day^{-1}	0.016	21.2 m^3 day^{-1}	0.021	**0.005**
Schaffer-nicht et al. 1995 – Anesthetists	Absorption fraction	27%	0.84	50%	1.56	**0.72**
	Metabolism fraction	12%	0.84	20%	1.41	**0.57**
	Inhalation rate	16 m^3 day^{-1}	0.84	21.2 m^3 day^{-1}	1.11	**0.27**
	Halothane concentration in air	380 mg m^{-3}	0.84	700 mg m^{-3}	2.05	**1.2**
Moore et al. 1986 – Acyanotic	Absorption fraction	27%	35.83	50%	66.40	**30.6**
	Metabolism fraction	12%	35.83	20%	59.70	**23.9**
	Inhalation rate	0.21 L kg^{-1} min^{-1}	35.83	0.31 L kg^{-1} min^{-1}	54.30	**18.5**
Bentley et al. 1981 – Obese	Absorption fraction	27%	9.13	50%	16.91	**7.8**
	Metabolism fraction	12%	9.13	20%	15.22	**6.1**
	Inhalation rate	0.12 L kg^{-1} min^{-1}	9.13	0.18 L kg^{-1} min^{-1}	13.70	**1.2**
Sutton et al 1991 – Volunteers	Absorption fraction	8%	2.60	NA		
	Metabolism fraction	0.002%	2.60	NA		
	Inhalation rate	0.12 L kg^{-1} min^{-1}	2.60	0.18 L kg^{-1} min^{-1}	3.91	**1.2**

Exposure estimation in several studies involved additional uncertainties. In Monte et al. (1994), urine samples were analyzed only up to 6 hours following exposure to HFA-134a. Given that the half-life of TFA has been found to be 42 hours when produced via halothane metabolism in children (Wark et al., 1990), this limited sampling period may have resulted in considerable underestimation of TFA exposure. Additionally, the detection limit was 10 ng/mL, which may have been insufficiently sensitive for identifying low TFA concentrations. In Schaffernicht et al. (1995), only the minimum and maximum concentrations of halothane in room air were reported. Consequently, mean concentration estimates relied solely on these two values and may not accurately reflect the true mean. Furthermore, no duration of exposure was given in this study, making the estimation of the total TFA dose very uncertain.

C2 - Quality review

JBI Critical Appraisal Checklist For Case Reports

Author: **Danielle H. Rochlin, MD, Charlotte M. Rajasingh, BA,Yvonne L. Karanas, MD, and Drew J. Davis, MD** Year: **2018**

	Yes	No	Unclear	Not applicable
1. Were patient's demographic characteristics clearly described?	□	X	□	□
2. Was the patient's history clearly described and presented as a timeline?	X	□	□	□
3. Was the current clinical condition of the patient on presentation clearly described?	X	□	□	□
4. Were diagnostic tests or assessment methods and the results clearly described?	X	□	□	□
5. Was the intervention(s) or treatment procedure(s) clearly described?	X	□	□	□
6. Was the post-intervention clinical condition clearly described?	X	□	□	□
7. Were adverse events (harms) or unanticipated events identified and described?	X	□	□	□
8. Does the case report provide takeaway lessons?	X	□	□	□

Overall appraisal: Include X Exclude □ Seek further info □

Comments (Including reason for exclusion)

Race of 23-year old woman not included. Report states TFA burns on 15% total body surface area (legs, buttocks) from spilling TFA during work and slipping onto it. Patient suffered from full-thickness burns.

JBI Critical Appraisal Checklist For Case Reports

Author: **Ji Yeon Byun, Ju Yun Woo, You Won Choi and Hae Young Choi** Year:_**2013**

	Yes	No	Unclear	Not applicable
1. Were patient's demographic characteristics clearly described?	X	□	□	□
2. Was the patient's history clearly described and presented as a timeline?	X	□	□	□
3. Was the current clinical condition of the patient on presentation clearly described?	X	□	□	□
4. Were diagnostic tests or assessment methods and the results clearly described?	X	□	□	□
5. Was the intervention(s) or treatment procedure(s) clearly described?	X	□	□	□
6. Was the post-intervention clinical condition clearly described?	X	□	□	□
7. Were adverse events (harms) or unanticipated events identified and described?	X	□	□	□
8. Does the case report provide takeaway lessons?	X	□	□	□

Overall appraisal: Include X Exclude □ Seek further info □

Comments (Including reason for exclusion)

Patient was a 24-year old Korean man that suffered from allergic reaction to TFA (and benzyl alcohol and phenoxyethanol). The study highlighted that the contact dermatitis occurred due to an allergic reaction rather than chemical irritation. Current clinical condition included recurring symptoms after 6 months after treatment with oral methylprednisolone and topical steroids after contact with these substances during his work in the laboratory.

JBI Critical Appraisal Checklist For Case Reports

Author: **Christie Sun, Bryan Corbett** Year: **2017**

	Yes	No	Unclear	Not applicable
9. Were patient's demographic characteristics clearly described?	□	X	□	□
10. Was the patient's history clearly described and presented as a timeline?	X	□	□	□

	Yes	No	Unclear	Not applicable
11. Was the current clinical condition of the patient on presentation clearly described?	X	□	□	□
12. Were diagnostic tests or assessment methods and the results clearly described?	X	□	□	□
13. Was the intervention(s) or treatment procedure(s) clearly described?	X	□	□	□
14. Was the post-intervention clinical condition clearly described?	X	□	□	□
15. Were adverse events (harms) or unanticipated events identified and described?	□	□	□	X
16. Does the case report provide takeaway lessons?	X	□	□	□

Overall appraisal: Include X Exclude □ Seek further info □

Comments (Including reason for exclusion)

Case reports does not include the race of the 27-year old male patient; no adverse effects were stated; patient was doing well after his s24-hour stay at the emergency unit. Patient received burns on right forearms from 1 L TFAA accidental spillage during work.

JBI Critical Appraisal Checklist For Case Reports

Author: **Christine Nguyen, Noel R. Rose, and Dolores B. Njoku**_Year: **2008**

	Yes	No	Unclear	Not applicable
17. Were patient's demographic characteristics clearly described?	X	□	□	□
18. Was the patient's history clearly described and presented as a timeline?	X	□	□	□
19. Was the current clinical condition of the patient on presentation clearly described?	X	□	□	□
20. Were diagnostic tests or assessment methods and the results clearly described?	X	□	□	□
21. Was the intervention(s) or treatment procedure(s) clearly described?	X	□	□	□
22. Was the post-intervention clinical condition clearly described?	X	□	□	□
23. Were adverse events (harms) or unanticipated events identified and described?	X	□	□	□
24. Does the case report provide takeaway lessons?	X	□	□	□

Overall appraisal: Include X Exclude □ Seek further info □

Comments (Including reason for exclusion)

4-year old obese hispanic girl suffered from acute liver failure attributed to halothance hepatitis – "current" clinical conidition of patient involved her discharge from hospital after 21 days post operation. Treatment with INHE 4 years prior + obesity are suspected to be causes for the halothance acute liver failure already after the first halothane exposure.

JBI Critical Appraisal Checklist For Case Reports

Author: **H. Wark, J. Earl and J. Overton** Year: **1991**

	Yes	No	Unclear	Not applicable
Were there clear criteria for inclusion in the case series?	□	□	X	□
Was the condition measured in a standard, reliable way for all participants included in the case series?	X	□	□	□
Were valid methods used for identification of the condition for all participants included in the case series?	X	□	□	□
Did the case series have consecutive inclusion of participants?	□	X	□	□
Did the case series have complete inclusion of participants?	□	X	□	□
Was there clear reporting of the demographics of the participants in the study?	□	X	□	□
Was there clear reporting of clinical information of the participants?	X	□	□	□
Were the outcomes or follow up results of cases clearly reported?	□	X	□	□
Was there clear reporting of the presenting site(s)/clinic(s) demographic information?	X	□	□	□
Was statistical analysis appropriate?	X	□	□	□

Overall appraisal: Include □ Exclude □ Seek further info X

Comments (Including reason for exclusion)

Case series did not provide a statement why these 2 patients were selected. Additionally, no mention of race was provided, only weight, age and gender of infants with their cause for admittance to the hospital (jaundice). Case series did not mention in which time frame patients were admitted and did not provide any information on the outcomes or any follow-up results of the cases.

JBI Critical Appraisal Checklist For Case Reports

Author: **Jakob Dahlin, Malin Engfeldt, Cecilia Svedman, Martin Mowitz, Erik Zimerson, Marle´ne Isaksson, Monica Hindse´n and Magnus Bruze** Year:**2013** Record Number: **doi:10.1111/cod.12059**

	Yes	No	Unclear	Not applicable
Were there clear criteria for inclusion in the case series?	X	□	□	□
Was the condition measured in a standard, reliable way for all participants included in the case series?	□	□	□	X
Were valid methods used for identification of the condition for all participants included in the case series?	□	□	□	X
Did the case series have consecutive inclusion of participants?	X	□	□	□
Did the case series have complete inclusion of participants?	X	□	□	□
Was there clear reporting of the demographics of the participants in the study?	□	X	□	□
Was there clear reporting of clinical information of the participants?	X	□	□	□
Were the outcomes or follow up results of cases clearly reported?	X	□	□	□
Was there clear reporting of the presenting site(s)/clinic(s) demographic information?	□	□	□	X
Was statistical analysis appropriate?	□	□	□	X

Overall appraisal: Include X Exclude □ Seek further info □

Comments (Including reason for exclusion): **Patients' race and education were not provided. As this case series only included chemical burns, no standard measurements of condition or identification of condition are applicable. All patients suffered from burns during their work with TFA in the laboratory. All burns improved over time.**

JBI Critical Appraisal Checklist For Case Reports

Author: **Perrine Hoet, Mary Louise M Graf, Mohammed Bourdi, Lance R Pohl, Paul H Duray, Weiqiao Chen, Raimund M Peter, Sidney D Nelson, Nicolas Verlinden, Dominique Lison** Year: **1997**

	Yes	No	Unclear	Not applicable
Were there clear criteria for inclusion in the case series?	X	□	□	□
Was the condition measured in a standard, reliable way for all participants included in the case series?	X	□	□	□
Were valid methods used for identification of the condition for all participants included in the case series?	□	□	X	□
Did the case series have consecutive inclusion of participants?	□	□	X	□
Did the case series have complete inclusion of participants?	□	□	X	□
Was there clear reporting of the demographics of the participants in the study?	□	□	X	□
Was there clear reporting of clinical information of the participants?	□	□	X	□
Were the outcomes or follow up results of cases clearly reported?	□	□	X	□
Was there clear reporting of the presenting site(s)/clinic(s) demographic information?	□	□	□	X
Was statistical analysis appropriate?	X	□	□	□

Overall appraisal: Include □ Exclude □ Seek further info X

Comments (Including reason for exclusion)

The case series described in detail the first patient's symptoms, diagnosis tools (blood work, biopsy) as well as the general trend of the disease and progression. However, patient 2-6 were barely described and it was not clear, what their outcomes were. Race of patients was also not mentioned.

Newcastle - Ottawa Quality Assessment Scale Cohort Studies

Author: **Bentley, J. B., Vaughan, R. W., Gandolfi, A. J., Cork, R. C.** Year: **1981**

Selection

1) Representativeness of the exposed cohort

a) truly representative of the average _______________ (describe) in the community

b) somewhat representative of the average ______________ in the community

c) selected group of users eg nurses, volunteers- ausgewähltes OP-personal, nicht sicher, ob repräsentativ für ihre Gruppe und nicht sicher wie ausgewählt

d) no description of the derivation of the cohort

2) Selection of the non exposed cohort – alle exponiert, vergleich zw. Op-Räumen

a) drawn from the same community as the exposed cohort

b) drawn from a different source

c) no description of the derivation of the non exposed cohort

3) Ascertainment of exposure

a) secure record (eg surgical records)

b) structured interview

c) written self report

d) no description

4) Demonstration that outcome of interest was not present at start of study

a) yes

b) no

Comparability

1) Comparability of cohorts on the basis of the design or analysis

a) study controls for __________ ___ (select the most important factor)

b) study controls for any additional factor (This criteria could be modified to indicate specific control for a second important factor.)

Outcome

1) Assessment of outcome

a) independent blind assessment

b) record linkage

c) self report

d) no description

2) Was follow-up long enough for outcomes to occur

a) yes (select an adequate follow up period for outcome of interest)

b) no

3) Adequacy of follow up of cohorts

a) complete follow up - all subjects accounted for

b) subjects lost to follow up unlikely to introduce bias - small number lost - > ____ % (select an adequate %) follow up, or description provided of those lost)

c) follow up rate < ____% (select an adequate %) and no description of those lost

d) no statement

Points:

1) 3 of 4 (1 criteriom not applicable)
2) 0 of 2
3) 3 of 3

Total: 6 of 9

Newcastle - Ottawa Quality Assessment Scale Cohort Studies

Author: **Takiyama, R., Morio, M., Fujii, K., Kikuchi, H., Yuge, O., Chikasue, F., Taira, Y., Jordanov, J. G.**Year: **1985**

Selection

1) Representativeness of the exposed cohort

a) truly representative of the average _______________ (describe) in the community

b) somewhat representative of the average ______________ in the community

c) selected group of users eg nurses, volunteers- ausgewählte Patienten, nicht sicher, ob repräsentativ für ihre Gruppe und nicht sicher wie ausgewählt

d) no description of the derivation of the cohort

2) Selection of the non exposed cohort – beide exposed

a) drawn from the same community as the exposed cohort

b) drawn from a different source

c) no description of the derivation of the non exposed cohort

3) Ascertainment of exposure

a) secure record (eg surgical records)

b) structured interview

c) written self report

d) no description

4) Demonstration that outcome of interest was not present at start of study

a) yes

b) no

Comparability

1) Comparability of cohorts on the basis of the design or analysis

a) study controls for __________health___ (select the most important factor)

b) study controls for any additional factor (This criteria could be modified to indicate specific control for a second important factor.)

Outcome

1) Assessment of outcome

a) independent blind assessment

b) record linkage

c) self report

d) no description

2) Was follow-up long enough for outcomes to occur

a) yes (select an adequate follow up period for outcome of interest)

b) no

3) Adequacy of follow up of cohorts

a) complete follow up - all subjects accounted for

b) subjects lost to follow up unlikely to introduce bias - small number lost - > ____ % (select an adequate %) follow up, or description provided of those lost)

c) follow up rate < ____% (select an adequate %) and no description of those lost

d) no statement

Points:

1) 2 of 4
2) 1 of 2
3) 3 of 3

Total: 6 of 9

Newcastle - Ottawa Quality Assessment Scale Cohort Studies

Author: **Wegner, R., Rincker, B., Poschadel, B., Szadkowski, D.** Year: **1990**

Selection

1) Representativeness of the exposed cohort

a) truly representative of the average _______________ (describe) in the community

b) somewhat representative of the average ______________ in the community

c) selected group of users eg nurses, volunteers- ausgewähltes OP-personal, nicht sicher, ob repräsentativ für ihre Gruppe und nicht sicher wie ausgewählt

d) no description of the derivation of the cohort

2) Selection of the non exposed cohort – alle exponiert, vergleich zw. Op-Räumen

a) drawn from the same community as the exposed cohort

b) drawn from a different source

c) no description of the derivation of the non exposed cohort

3) Ascertainment of exposure

a) secure record (eg surgical records)

b) structured interview

c) written self report

d) no description

4) Demonstration that outcome of interest was not present at start of study

a) yes

b) no

Comparability

1) Comparability of cohorts on the basis of the design or analysis

a) study controls for __________ ___ (select the most important factor)

b) study controls for any additional factor (This criteria could be modified to indicate specific control for a second important factor.)

Outcome

1) Assessment of outcome

a) independent blind assessment

b) record linkage

c) self report

d) no description

2) Was follow-up long enough for outcomes to occur

a) yes (select an adequate follow up period for outcome of interest)

b) no

3) Adequacy of follow up of cohorts

a) complete follow up - all subjects accounted for

b) subjects lost to follow up unlikely to introduce bias - small number lost - > ____ % (select an adequate %) follow up, or description provided of those lost)

c) follow up rate < ____% (select an adequate %) and no description of those lost

d) no statement

Points:

1) 3 of 4 (1 criteriom not applicable)
2) 0 of 2
3) 3 of 3

Total: 6 of 9

Newcastle - Ottawa Quality Assessment Scale Cohort Studies

Author: **Moore, R. A., McNicholas, K. W., Gallagher, J. D., Gandolfi, A. J., Sipes, I. G., Kerns, D., Clark, D. L.** Year: **1986**

Selection

1) Representativeness of the exposed cohort

a) truly representative of the average _______________ (describe) in the community

b) somewhat representative of the average ______________ in the community

c) selected group of users eg nurses, volunteers- ausgewähltes OP-personal, nicht sicher, ob repräsentativ für ihre Gruppe und nicht sicher wie ausgewählt

d) no description of the derivation of the cohort

2) Selection of the non exposed cohort – alle exponiert, vergleich zw. Sauerstofflevel

a) drawn from the same community as the exposed cohort

b) drawn from a different source

c) no description of the derivation of the non exposed cohort

3) Ascertainment of exposure

a) secure record (eg surgical records)

b) structured interview

c) written self report

d) no description

4) Demonstration that outcome of interest was not present at start of study

a) yes

b) no

Comparability

1) Comparability of cohorts on the basis of the design or analysis

a) study controls for ___________ ___ (select the most important factor)

b) study controls for any additional factor (This criteria could be modified to indicate specific control for a second important factor.)

Outcome

1) Assessment of outcome

a) independent blind assessment

b) record linkage

c) self report

d) no description

2) Was follow-up long enough for outcomes to occur

a) yes (select an adequate follow up period for outcome of interest)

b) no

3) Adequacy of follow up of cohorts

a) complete follow up - all subjects accounted for

b) subjects lost to follow up unlikely to introduce bias - small number lost - > ____ % (select an adequate %) follow up, or description provided of those lost)

c) follow up rate < ____% (select an adequate %) and no description of those lost

d) no statement

Points:

1) 2 of 4 (1 criteriom not applicable)
2) 0 of 2
3) 3 of 3

Total: 5 of 9

Newcastle - Ottawa Quality Assessment Scale Cohort Studies

Author: **Sutton, T. S., Koblin, D. D., Gruenke, L. D., Weiskopf, R. B., Rampil, I. J., Waskell, L., Eger, E. I., 2nd** Year: **1991**

Selection

1) Representativeness of the exposed cohort

a) truly representative of the average _______________ (describe) in the community

b) somewhat representative of the average ______________ in the community

c) selected group of users eg nurses, volunteers- ausgewählte Patienten, nicht sicher, ob repräsentativ für ihre Gruppe und nicht sicher wie ausgewählt

d) no description of the derivation of the cohort

2) Selection of the non exposed cohort – Exposition unbekannt

a) drawn from the same community as the exposed cohort

b) drawn from a different source

c) no description of the derivation of the non exposed cohort

3) Ascertainment of exposure

a) secure record (eg surgical records)

b) structured interview

c) written self report

d) no description

4) Demonstration that outcome of interest was not present at start of study

a) yes

b) no

Comparability

1) Comparability of cohorts on the basis of the design or analysis

a) study controls for __________BMI, Glucose, demographic___ (select the most important factor)

b) study controls for any additional factor (This criteria could be modified to indicate specific control for a second important factor.)

Outcome

1) Assessment of outcome

a) independent blind assessment

b) record linkage

c) self report

d) no description

2) Was follow-up long enough for outcomes to occur

a) yes (select an adequate follow up period for outcome of interest)

b) no

3) Adequacy of follow up of cohorts

a) complete follow up - all subjects accounted for

b) subjects lost to follow up unlikely to introduce bias - small number lost - > ____ % (select an adequate %) follow up, or description provided of those lost)

c) follow up rate < ____% (select an adequate %) and no description of those lost

d) no statement

Points:

1) 0 of 4 (1 criteriom not applicable)
2) 1 of 2
3) 3 of 3

Total: 4 of 9
